# In Vitro Antifungal Efficacy of Blue-Light Photodynamic Therapy with Curcumin and Riboflavin Formulation Activated by 450 nm Diode Laser Against *Candida albicans* Biofilm on Titanium Implants

**DOI:** 10.3390/pharmaceutics17111437

**Published:** 2025-11-07

**Authors:** Aleksandra Warakomska, Małgorzata Kępa, Jakub Fiegler-Rudol, Katarzyna Latusek-Kotyczka, Dariusz Skaba, Rafał Wiench

**Affiliations:** 1 Department of Periodontal Diseases and Oral Mucosa Diseases, Faculty of Medical Sciences in Zabrze, Medical University of Silesia, 40-055 Katowice, Poland; s88998@365.sum.edu.pl (J.F.-R.); katarzyna.latusek@sum.edu.pl (K.L.-K.); rwiench@sum.edu.pl (R.W.); 2 Department of Microbiology, Faculty of Pharmaceutical Sciences in Sosnowiec, Medical University of Silesia, Jagiellońska 4, 41-200 Sosnowiec, Poland; mkepa@sum.edu.pl

**Keywords:** antimicrobial photodynamic therapy, blue laser, curcumin, peri-implantitis, riboflavin

## Abstract

**Background**: *Candida albicans* is increasingly recognized in peri-implantitis due to its capacity to form resilient biofilms on implant surfaces, limiting treatment success. Antimicrobial photodynamic therapy (aPDT) may offer a non-invasive adjunct by leveraging photosensitizer activation to produce reactive oxygen species that disrupt microbial cells. This in vitro study assessed the antifungal efficacy of QroxB2, a dual-photosensitizer containing riboflavin and curcumin, activated by 450 nm blue light against *C. albicans* biofilms on titanium implants. **Methods**: *C. albicans* biofilms were formed on 63 titanium implants and randomly assigned to nine groups (*n* = 7): untreated control (GC), chlorhexidine (CHX), riboflavin (RIB), curcumin (CUR), QroxB2 (QBX), laser only (L), and three photodynamic therapy groups combining laser irradiation with each photosensitizer (L + RIB, L + CUR, L + QBX). Treatments were followed by colony-forming unit (CFU) enumeration. **Results**: The L + QBX group showed the strongest antifungal effect, achieving a 94% reduction in fungal load, with median CFU counts decreasing from 49,000 in the untreated control to 2800 CFU/mL. CHX eradicated all viable cells (0 CFU/mL). Among photosensitizer-only groups, QBX produced a moderate reduction (median 21,800 CFU/mL), whereas laser irradiation alone (L) exhibited no meaningful antifungal activity, with median counts comparable to the untreated control (49,000 CFU/mL). **Conclusions**: QroxB2-mediated aPDT achieved a significant reduction in *Candida albicans* colony-forming units on implant surfaces. While not as potent as chlorhexidine, this light-activated, biocompatible approach may serve as a complementary tool in managing peri-implant fungal infections. Clinical validation is warranted.

## 1. Introduction

### 1.1. Background and Epidemiology

Dental implants are widely used for tooth replacement, with about 18 million placed annually [1]. Their success, however, is increasingly challenged by peri-implantitis, which affects on average 22% of patients [2] and is projected to rise by 2030 [3]. Risk factors include periodontal disease, osteoporosis, and bruxism [4]. The condition is primarily associated with anaerobic bacteria, notably Socransky’s red complex species such as *Porphyromonas gingivalis*, *Tannerella forsythia*, and *Treponema denticola* [5,6,7]. Although peri-implantitis is primarily driven by anaerobic bacteria, emerging evidence indicates that yeasts, particularly *Candida albicans*, contribute to disease persistence and treatment resistance. Their ability to adhere to titanium, form resilient biofilms, and coexist synergistically with bacterial pathogens suggests that antifungal strategies may be necessary to complement conventional antibacterial approaches [1,2,3,4,5,6,7,8,9,10]. More recent evidence also implicates yeasts, particularly *Candida albicans*, in peri-implant lesions [8,9], broadening the ecological model of disease.

### 1.2. Role of Candida albicans

Clinically, peri-implantitis is defined by inflammation and progressive bone loss [10], often following peri-implant mucositis, which presents without bone loss [10,11,12]. Management involves non-surgical debridement with ultrasonic or non-metallic instruments, sometimes supported by adjuncts such as chlorhexidine, antibiotics, or antimicrobial photodynamic therapy (aPDT) [11,12,13,14,15,16]. Surgical interventions may also incorporate aPDT [17]. This approach combines a photosensitizer, a light source, and oxygen to generate reactive oxygen species (ROS) that oxidize microbial lipids, proteins, and nucleic acids [18,19,20,21,22,23]. In *Candida albicans*, ROS also impair mitochondria, disrupt cell wall integrity, and induce apoptosis-like responses [24]. Both natural and synthetic photosensitizers are available [25]. Notably, curcumin and riboflavin activated by 450 nm blue light show antifungal activity in vitro, including against *C. albicans* [24,25,26,27]. Still, aPDT is less effective against biofilms than planktonic cells [18,28]; biofilm-associated microorganisms, including *Candida* with its larger, structurally complex cells, are more resistant [18,29]. While both curcumin and riboflavin have been studied individually as photosensitizers against planktonic *Candida* cells and, to a lesser extent, biofilms, most investigations have tested them in isolation, under variable light conditions, and rarely on titanium implant surfaces.

### 1.3. Rationale for aPDT

Previous reports confirmed that curcumin-mediated aPDT can suppress *Candida*, impairing cellular metabolic processes, and that riboflavin-mediated aPDT may eradicate biofilms under specific settings. However, the combination of curcumin and riboflavin in a single formulation has not been systematically evaluated against mature *Candida albicans* biofilms on implant-grade titanium. This dual system is expected to offer complementary photochemical mechanisms: curcumin favoring type I radical pathways, and riboflavin favoring type II singlet oxygen generation, which may enhance efficacy in the oxygen-limited and matrix-rich biofilm microenvironment. By applying a clinically accessible 450 nm diode laser, our study specifically addresses whether a curcumin–riboflavin dual photosensitizer can overcome the inherent resistance of *Candida* biofilms on implant surfaces. This is the first report directly testing such a formulation against *Candida albicans* biofilm on titanium implants, establishing a rationale for future translational work in peri-implantitis management. While the present protocol was developed as an in vitro model, it simulates peri-implant surface contamination by *Candida albicans* and allows controlled evaluation of antifungal strategies on implant surfaces, with potential translational relevance for peri-implantitis management.

### 1.4. Objectives

Curcumin absorbs mainly in the blue range (~430 nm) and tends to mediate radical-based Type I photodynamic effects. Riboflavin absorbs near UV and blue (375–445 nm) and favors Type II singlet oxygen generation, though it can also produce radicals depending on oxygen levels. Both short-wavelength-absorbing photosensitizers are used for their biocompatibility and dual reactive oxygen species generation [26,27,28,29,30,31,32,33,34,35,36].

## 2. Materials and Methods

The study was conducted at the Department of Microbiology, Faculty of Pharmaceutical Sciences of Medical University of Silesia, Sosnowiec, Poland.

### 2.1. Reference Microbial Strains

The experiment utilized the reference strain *Candida albicans* ATCC 10231, obtained from the American Type Culture Collection (ATCC, Manassas, VA, USA). We cultured the strain on Sabouraud Dextrose Agar (SDA; bioMérieux, Marcy-l’Étoile, France) and incubated at 37 °C for 24 h. Approximately five colonies (≥1 mm) were suspended in physiological saline and adjusted to a 0.5 McFarland standard (1 to 5 × 10^6^ colony-forming units per mL, CFU/mL), verified with a Densi-La-Meter II densitometer (Erba Polska, Kraków, Poland).

### 2.2. Laser and Photosensitizer

In this study, three photosensitizing formulations were used: QroxB2, Curcumin Gel 95+, and Xlinker Gel.

QroxB2 (CMSDental, Roslev, Denmark) was used as a reference photosensitizer, primarily marketed for antimicrobial photodynamic applications in dentistry. The product is supplied as a yellowish, dry powder in a single-use syringe and is reconstituted with sterile, demineralized water immediately before use, forming a viscous gel. The exact concentrations of its active components are not disclosed in the publicly available specifications. It is sold in two presentation volumes, 0.5 mL (QroxB2 Endo) and 1.5 mL (QroxB2 Perio), depending on the clinical indication.

Curcumin-Gel95+ (BioenergeticLab, Brescia, Italy) is a commercially available gel formulation containing two standardized Curcuma longa L. rhizome extracts titrated to 20% and 47.5% curcuminoids, respectively. Each 5 mL stick-pack contains 83.5 mg of total curcuminoids (36 mg from the 20% extract and 47.5 mg from the 47.5% extract). The gel base consists of water, β-cyclodextrin, xanthan gum, citric acid, potassium sorbate, and sucralose. The formulation is designed as a food-grade hydrogel for oral administration; however, its physicochemical properties make it suitable for experimental photodynamic applications on solid surfaces. The total curcuminoid concentration in the product corresponds approximately to 16.7 mg/mL (83.5 mg per 50 mL portion). The product was stored at < 25 °C, protected from light, and freshly dispensed from sealed stick-packs immediately before each application to minimize photodegradation.

Xlinker Gel (Ophtechnics Unlimited, Haryana, India) contains 0.1% *w*/*v* riboflavin sodium phosphate and 20% *w*/*v* dextran in a sterile aqueous base (single-use vial, 3 mL). The product’s original indication is for corneal collagen crosslinking in ophthalmology, but its physicochemical characteristics make it compatible with blue-light APDT.

Irradiation was carried out using a 450 nm diode laser (PIOON S1 Blue, PIOON, Wuhan, China) operating in continuous wave (CW) mode. The laser was equipped with a flat-top handpiece, ensuring uniform energy distribution across the beam profile and eliminating hot-spot formation commonly associated with Gaussian power distributions. The applicator diameter was 8 mm, corresponding to an irradiated area of ~0.5 cm^2^. The tip was positioned 2 mm from the surface. Power was 400 mW for 120 s per surface, giving 96 J/cm^2^ at 0.8 W/cm^2^.

### 2.3. Experimental Design 

Sixty-three brand-new, sterile dental implants (Keystone Dental, 3.5 × 10 mm, Burlington, MA, USA) with a sand-blasted, acid-etched surface were used. No additional coatings were present, and surface roughness was standardized across all implants as supplied by the manufacturer. Implants were provided in manufacturer-sealed sterile packaging and opened immediately prior to use under aseptic conditions. No implants were reused across experiments.

The tested implants were individually suspended on sterile threads and infected with *C. albicans* biofilms. Implants were individually placed into 50 mL Falcon tubes containing 20 mL of sterile Sabouraud Dextrose Broth. To each tube, 1 mL of *C. albicans* (ATCC 10231) suspension adjusted to a 0.5 McFarland standard was added. Implants were positioned to ensure complete immersion in the inoculum. Incubation was carried out under aerobic conditions at 37 °C on an orbital shaker set to 150 rpm for 96 h, allowing biofilm formation. This model simulates the secondary colonization of dental implants observed in peri-implantitis, enabling standardized testing of antifungal interventions under reproducible conditions. After incubation, implants were randomly assigned to nine groups (n = 7 per group). The following groups were analyzed:

- GC (Growth Control): No treatment applied.
- CHX: Treated with 1% chlorhexidine gluconate gel for 2 min.
- RIB: Treated with riboflavin gel (Xlinker Gel) for 20 min in the dark.
- CUR: Treated with curcumin gel (Curcumin-Gel 95+) for 20 min in the dark.
- QBX: Treated with QroxB2 gel for 20 min in the dark.
- L: Exposed to laser light only (450 nm, 400 mW, 2 min per surface), without any photosensitizer.
- L + RIB: Treated with riboflavin gel for 20 min, rinsed with 0.9% NaCl, and then irradiated as above.
- L + CUR: Treated with curcumin gel for 20 min, rinsed with 0.9% NaCl, and then irradiated as above.
- L + QBX: Treated with QroxB2 for 20 min, rinsed with 0.9% NaCl, and then irradiated as above.

The 20 min incubation period was selected based on prior optimization studies involving curcumin–riboflavin photodynamic therapy, which demonstrated maximal antifungal activity at this pre-irradiation interval [37]. The same incubation time was applied to all photosensitizers (QroxB2, Curcumin Gel 95+, and Xlinker Gel) to ensure comparable adsorption kinetics and surface contact times, thereby minimizing methodological variability among formulations with differing viscosity and chromophore properties. All incubation steps were performed under light-protected conditions (opaque box, closed lid) at room temperature. Following incubation, rinsing with 0.9% NaCl was standardized to three washes of 1 mL each, performed immediately before irradiation to minimize variation in residual photosensitizer.

Following treatment, each implant was rinsed three times with sterile phosphate-buffered saline (PBS) and transferred into sterile 2.0 mL Eppendorf tubes containing 2 mL of sterile 0.9% saline solution. The tubes were vortexed for 1 min using a vortex mixer (Labnet Orbit 300, Labnet International, Edison, NJ, USA), sonicated in an ultrasonic bath for 5 min at 45 kHz (QUIQQ MD 18061, Medion, Essen, Germany), and vortexed again for 1 min to dislodge the biofilm from the implant surface. 1 mL of the resulting suspension was withdrawn and mixed with 1 mL of sterile saline. Subsequently, serial tenfold dilutions were prepared by transferring 1 mL of the previous dilution into 9 mL of sterile saline, resulting in dilution levels of 10^−1^, 10^−2^, and 10^−3^.

From each dilution, including the undiluted sample (10^0^), 100 μL was surface plated onto Sabouraud Dextrose Agar using a calibrated 4 mm diameter loop, following the Hoeprich method. The plates were incubated at 37 °C for 24 h, after which colonies were counted on plates containing 30–300 CFUs, and the results are expressed as CFU/mL.

CFU/mL was calculated using the formula: CFU/mL = (number of colonies × dilution factor)/plated volume (mL), where only plates with 30–300 colonies were included to ensure accuracy.

### 2.4. Microbiological Analysis

The primary outcome parameter was the total yeast count, expressed as colony-forming units per milliliter (CFU/mL), determined according to internal laboratory standards. Each treatment group consisted of seven independently prepared implants (*n* = 7), serving as true biological replicates, as biofilms were cultivated separately on each implant. For CFU enumeration, one measurement was obtained per implant after biofilm dislodgement.

### 2.5. Nanocarrier Characterization

QroxB2 was characterized prior to experimental use. Particle size distribution and polydispersity index (PDI) were determined by dynamic light scattering (DLS) using a Zetasizer Nano ZS (Malvern Instruments, Worcestershire, UK) after reconstitution in sterile demineralized water (*n* = 3 independent preparations). ζ-potential was measured to assess surface charge and colloidal stability. Stability was monitored over 7 days at 4 °C and 37 °C by repeated DLS and ζ-potential measurements. Release kinetics of curcumin and riboflavin were evaluated under physiologically relevant conditions (PBS, pH 7.4, 37 °C) using a dialysis membrane method (molecular weight cutoff: 12–14 kDa). Aliquots were collected at predefined time points up to 48 h, and photosensitizer concentrations were quantified by UV–Vis spectroscopy at 420 nm (curcumin) and 445 nm (riboflavin). Data are expressed as mean ± SD from three independent experiments.

Curcumin Gel 95+ and Xlinker Gel, are commercially available gels with well-defined and manufacturer-validated compositions. Due to their semisolid matrix structure and manufacturer-defined formulation properties, further physicochemical characterization, such as DLS or ζ-potential analysis, was not applicable or required for this study.

### 2.6. Statistical Analysis

Power analysis was conducted prior to the study. The required sample size (*n* = 7) was estimated using Cohen’s formula [38]. Group differences in CFU/mL were assessed using the Kruskal–Wallis test followed by post hoc pairwise comparisons. All statistical analyses were performed in GraphPad Prism version 10 (GraphPad Software, San Diego, CA, USA), with *p*-values < 0.05 considered statistically significant.

## 3. Results

Descriptive statistics for total yeast load (CFU/mL) across all experimental groups are presented in Table 1.

Among the analyzed groups, the highest values were recorded in the GC group (median = 49,000 CFU/mL; range = 23,600–71,000) and in the laser-only group (L) (median = 49,000 CFU/mL; range = 13,300–69,000), both showing minimal or no reduction in viable cells compared with the untreated control. The RIB group showed a similar level of colonization (median = 45,100 CFU/mL; range = 21,700–75,200), while the CUR group exhibited a moderate reduction (median = 32,700 CFU/mL; range = 10,300–60,700). The QBX group demonstrated a stronger antifungal effect (median = 21,800 CFU/mL; range = 8300–84,000), corresponding to a 55.5% reduction relative to the control.

Photodynamic protocols produced further decreases in CFU counts. The L + RIB group reached a median of 33,000 CFU/mL (range = 10,850–45,500), whereas L + CUR and L + QBX achieved markedly lower values, with medians of 9850 CFU/mL (range = 3100–11,500) and 2800 CFU/mL (range = 1500–5000), respectively. The CHX group showed complete eradication of viable cells (0 CFU/mL).

**Figure 1:**
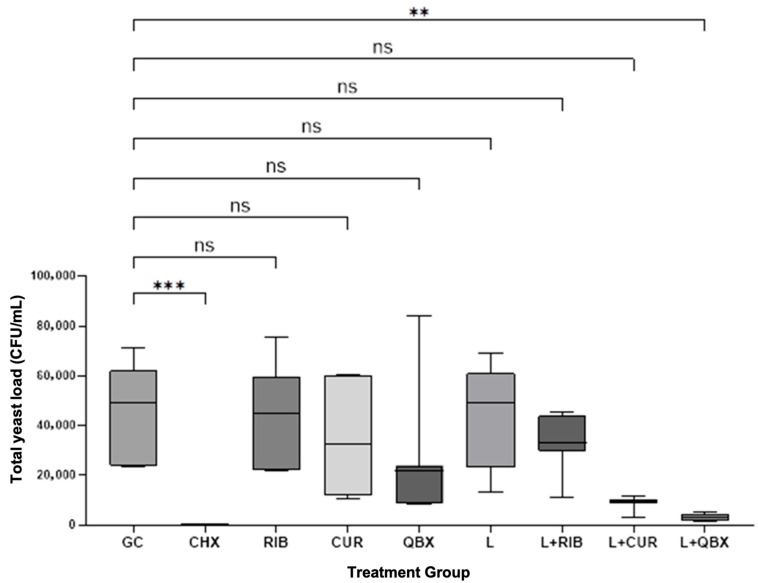
Comparison of total yeast load Colony Forming Units per milliliter (CFU/mL) between the growth control (GC) and treatment groups. Statistical comparisons were performed using the Kruskal–Wallis test followed by Dunn’s post hoc test. Significant differences vs. GC are marked: *p* < 0.01, *p* < 0.001; ns—not significant. CHX—Chlorhexidine; RIB—Xlinker Gel (riboflavin 0.1%); CUR—Curcumin-Gel 95+; QBX—QroxB2; L—Laser irradiation only; L + RIB—Xlinker Gel + Laser (riboflavin-mediated antimicrobial photodynamic therapy); L + CUR—Curcumin-Gel 95+ + Laser (curcumin-mediated antimicrobial photodynamic therapy); L + QBX—QroxB2 + Laser (QroxB2-mediated antimicrobial photodynamic therapy). ns = not significant ** = *p* < 0.01 *** = *p* < 0.001.

**Figure 2:**
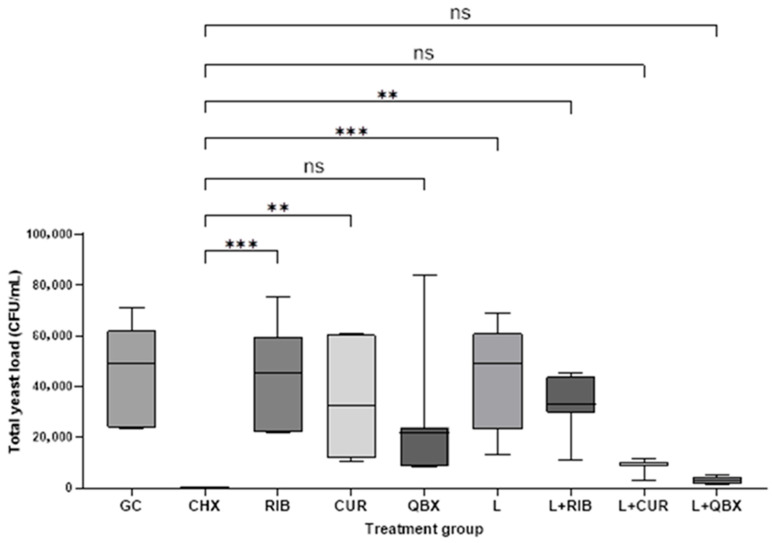
Comparison of total yeast load in Colony Forming Units per milliliter (CFU/mL) between treatment groups and the chlorhexidine group (CHX). Statistical comparisons were performed using the Kruskal–Wallis test followed by Dunn’s post hoc test. Significant differences vs. GC are marked: *p* < 0.01, *p* < 0.001. GC—Growth Control; RIB—Xlinker Gel (riboflavin 0.1%); CUR—Curcumin-Gel 95+; QBX—QroxB2; L—Laser irradiation only; L + RIB—Xlinker Gel + Laser (riboflavin-mediated antimicrobial photodynamic therapy); L + CUR—Curcumin-Gel 95+ + Laser (curcumin-mediated antimicrobial photodynamic therapy); L + QBX—QroxB2 + Laser (QroxB2-mediated antimicrobial photodynamic therapy). ns = not significant ** = *p* < 0.01 *** = *p* < 0.001.

**Figure 3:**
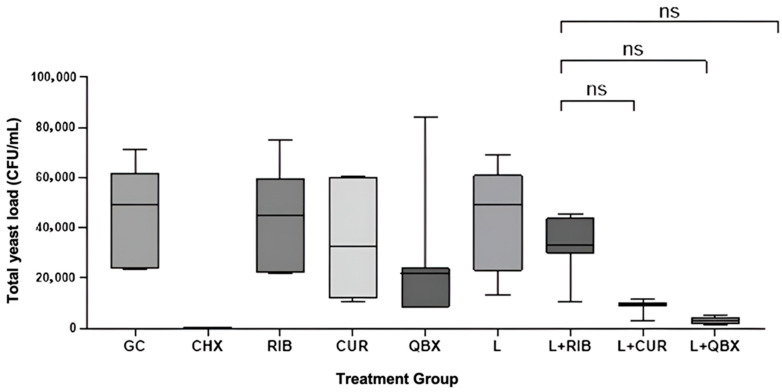
Comparison of total yeast load in Colony Forming Units per milliliter (CFU/mL) among antimicrobial photodynamic therapy groups. Statistical comparisons were performed using the Kruskal–Wallis test followed by Dunn’s post hoc test; ns—not significant. GC—Growth Control; CHX—Chlorhexidine; RIB—Xlinker Gel (riboflavin 0.1%); CUR—Curcumin-Gel 95+; QBX—QroxB2; L—Laser irradiation only; L + RIB—Xlinker Gel + Laser (riboflavin-mediated antimicrobial photodynamic therapy); L + CUR—Curcumin-Gel 95+ + Laser (curcumin-mediated antimicrobial photodynamic therapy); L + QBX—QroxB2 + Laser (QroxB2-mediated antimicrobial photodynamic therapy). ns = not significant.

The Kruskal–Wallis test confirmed statistically significant differences among the nine experimental groups (H = 44.48, *p* < 0.0001). Compared with the growth control (GC), a significant reduction in CFU/mL was observed only for CHX (*p* = 0.0004) and the photodynamic group using QroxB2 (L + QBX; *p* = 0.0157). No statistically significant differences were found between the GC group and the remaining groups (Figure 1). Compared with CHX, significantly higher CFU/mL values were observed in the GC, RIB, CUR, L, and L + RIB groups (*p* < 0.05). In contrast, no statistically significant differences were found between CHX and the QBX, L + CUR, or L + QBX groups (*p* > 0.13, Figure 2). No statistically significant differences were observed among the photodynamic therapy groups (L + RIB, L + CUR, and L + QBX) (*p* > 0.95, Figure 3). Although the L + QBX group achieved the highest mean CFU/mL reduction (94.3%), this improvement did not reach statistical significance compared to L + CUR or L + RIB.

**Figure 4:**
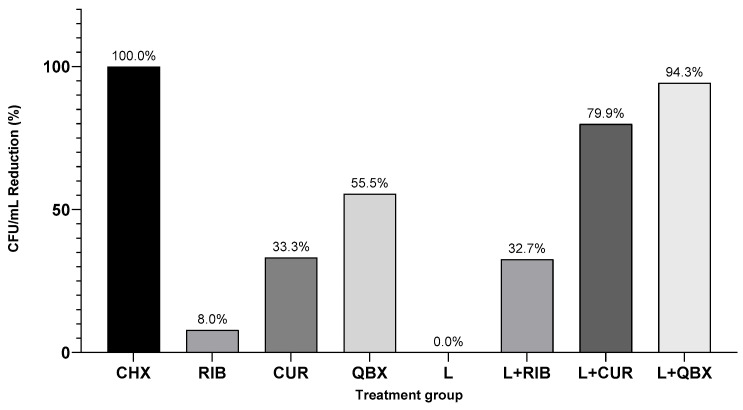
Median-based percentage reduction in Colony Forming Units per milliliter for each treatment group compared to the growth control (GC). CHX—Chlorhexidine; RIB—Xlinker Gel (riboflavin 0.1%); CUR—Curcumin-Gel 95+; QBX—QroxB2; L—Laser irradiation only; L + RIB—Xlinker Gel + Laser (riboflavin-mediated antimicrobial photodynamic therapy); L + CUR—Curcumin-Gel 95+ + Laser (curcumin-mediated antimicrobial photodynamic therapy); L + QBX—QroxB2 + Laser (QroxB2-mediated antimicrobial photodynamic therapy).

When expressed as a percentage reduction relative to the GC group, the following decreases in total yeast load were observed: CHX—100%, L + QBX—94.3%, L + CUR—79.9%, QBX—55.5%, CUR—33.3%, L + RIB—32.7%, RIB—8%, and L—0% (Figure 4).

## 4. Discussion

### 4.1. Significance of the Results

The central finding is that a dual curcumin–riboflavin photosensitizer activated at 450 nm reduced *Candida albicans* biofilm burden on titanium by 94%, positioning blue-light aPDT as a plausible adjunct where mechanical or chemical decontamination alone is insufficient. Chlorhexidine eradicated all viable cells in vitro, but its known cytotoxicity and wound-healing concerns constrain peri-implant use; by contrast, light-activated natural photosensitizers enable spatial control and may offer a more favorable therapeutic index in vivo. Against prior work, our effect size is competitive with or exceeds single-agent systems under comparable conditions. Curcumin at 40 μM with a 455 nm LED achieved more than 80% metabolic inhibition [30,39], and curcumin derivatives with erythrosine and nano-titanium dioxide produced about 1.1 log10 CFU/mL reductions on titanium [31]. Riboflavin at 0.1% with 455 nm LED reached complete biomass reduction on titanium [32], and riboflavin with UVA showed activity comparable to fluconazole against non-albicans species [33]. The present dual system approached riboflavin’s best-case outcomes without liposomal or polymeric encapsulation, suggesting that combining two chromophores can match or outperform nanoparticle carriers that typically report 70 to 90% inhibition depending on dose and wavelength [30,31,32,33]. This speaks to a formulation strategy that prioritizes complementary photochemistry over complex delivery vehicles, which may simplify translation.

Riboflavin-, curcumin-, and QroxB2-only groups (RIB, CUR, QBX) exhibited moderate CFU reductions compared with the growth control, consistent with the reported dark bioactivity of these chromophores on *Candida* membranes and metabolism. Such effects are typically attributed to non-photochemical mechanisms, including disruption of membrane integrity, interference with ergosterol biosynthesis, and ATPase inhibition [40,41,42,43]. However, these photosensitizer-only treatments did not differ statistically from the control, indicating that illumination was required to achieve reproducible decontamination. The most pronounced reduction occurred in the light-activated groups (L + RIB, L + CUR, and particularly L + QBX), supporting a light-dependent photodynamic component of action. Among them, only L + QBX differed significantly from the growth control, underscoring that the dual activation step was essential for reaching the highest efficacy within this experimental setting.

The biofilm context is essential for interpreting effect size and for benchmarking claims. Mature *C. albicans* biofilms comprise yeast, pseudohyphal, and hyphal forms, with hyphae conferring architectural stability and virulence [35] and an extracellular matrix that impedes antimicrobials [36]. aPDT routinely performs better against planktonic cells than biofilms [18,28,29,34]. Tkaczyk et al. reported up to 96% reductions for planktonic *Candida* with this dual system but lower effects on biofilms [37], a gradient mirrored here. That our protocol still achieved 94% CFU reduction on titanium, using a clinically accessible 450 nm diode and a 20 min preincubation derived from prior optimization [37], indicates that dual-photosensitizer blue-light aPDT can approach the upper bound reported for biofilm photoinactivation without specialized nanocarriers. Although blue light has limited tissue penetration and is more prone to scattering than longer wavelengths, our experimental model was confined to superficial biofilms on implant surfaces, where deep light propagation is not essential. Moreover, diode lasers operating at 445–450 nm have recently gained clinical relevance due to their high absorption in melanin and hemoglobin, as well as their low thermal thresholds, which enable effective biofilm targeting without extensive collateral heating. This wavelength range demonstrated antimicrobial effects, including against *C. albicans*, particularly when combined with riboflavin [38]. These lasers are currently available as compact, chairside-compatible devices for dental procedures, especially for surface-level disinfection strategies. Collectively, these factors support the translational rationale for using 450 nm aPDT in this context and justify its evaluation in the present in vitro study.

CFU enumeration was employed as the primary and most reliable endpoint for assessing fungal survival and treatment efficacy. The consistency of CFU results across groups, together with literature-aligned mechanisms [30,31,32,33,40,41,42,43,44,45,46,47,48,49,50], supports the conclusion that dual curcumin–riboflavin aPDT meaningfully disrupts fungal biofilms on implant-grade titanium, while also defining the boundaries of inference and the most reliable efficacy metric.

The curcumin–riboflavin combination may enhance biocompatibility by enabling antifungal effects at lower doses/fluence through complementary type I and II ROS pathways, reducing off-target exposure. Both compounds are food-derived and generally recognized as safe, with better safety profiles than many synthetic photosensitizers [25,40,41,42,43]. Under blue excitation, curcumin has been reported to favor electron or hydrogen transfer reactions typical of Type I pathways, and riboflavin to efficiently generate singlet oxygen typical of Type II, with cross-over possible depending on oxygen tension and microenvironment [25,26,27,28,41,42,43]. Our study did not measure ROS species directly, so we interpret the L + P+ effect as compatible with photochemical activation of the formulation rather than as proof of specific ROS pathways.

Both curcumin and riboflavin are photosensitizers that absorb light at relatively short wavelengths, mostly in the blue and near-UV spectrum (curcumin around 300–500 nm with a peak near 430 nm; riboflavin at approximately 375–380 nm and 445 nm) [26,27,28]. Shorter wavelengths such as blue light are more readily scattered, which reduces tissue penetration depth compared with photosensitizers that absorb at longer wavelengths in the red or near-infrared range. However, in the present in vitro setting, where the treatment target is a superficial biofilm on an exposed implant surface, deep light penetration is not required. Blue-absorbing photosensitizers remain practical because 450 nm diode lasers are compact, widely available in dental practice, and deliver uniform, surface-confined illumination with limited heating. Curcumin and riboflavin were chosen not only for their strong absorption at these wavelengths but also for their natural origin, safety, and biocompatibility [25,26,27,28]. Regarding the photochemical pathways, curcumin generally favors radical (Type I) mechanisms, in which the excited photosensitizer transfers electrons or hydrogen atoms to generate free radicals that subsequently form reactive oxygen species (ROS) and induce oxidative damage. Riboflavin, in contrast, is a well-established Type II photosensitizer that efficiently produces singlet oxygen (^1^O_2_) under fully aerated conditions, while it can also participate in Type I reactions under low-oxygen environments. These mechanistic distinctions are supported by studies employing quencher assays and ROS-specific probes in biochemical and microbiological systems, including Candida [25,41,42,43,44,45,46,47,48,49,50,51,52,53,54,55,56,57]. Using a mixture such as QroxB2 that contains both curcumin and riboflavin provides a rationale for exploiting complementary photochemical modes: curcumin contributing mainly to Type I radical formation and riboflavin to Type II singlet-oxygen generation. This combination may broaden the oxidative stress spectrum and enhance overall photodynamic efficacy against biofilm-associated fungi [26,27,28]. Nevertheless, the proprietary nature of QroxB2, with undisclosed proportions of its components, limits mechanistic resolution and standardization. We addressed this limitation by standardizing incubation, rinsing, irradiance, and energy density per surface area. Future studies should investigate defined ratios of these chromophores and compare their performance with longer-wavelength sensitizers that offer deeper tissue penetration while maintaining biocompatibility.

### 4.2. Clinical Relevance

Peri-implantitis care relies on reliable decontamination of rough titanium where mixed biofilms persist despite mechanical debridement and where chemical adjuncts such as chlorhexidine, although potent in vitro, can delay healing and irritate peri-implant tissues. The present data show that a blue-light aPDT protocol using curcumin and riboflavin reduced *Candida albicans* biofilm by 94% on implant-grade titanium, a performance that is competitive with single-agent photosensitizers and comparable to more complex nanocarriers, yet achieved with a simple formulation and a chairside-available 450 nm diode source. Clinically, this enables a targeted, surface-confined antimicrobial step that can be layered onto standard debridement without systemic exposure, with three immediate advantages for practice: first, spatial selectivity and short treatment times fit routine peri-implant maintenance or surgical access; second, broad oxidative killing can address fungal persistence that contributes to refractory disease alongside anaerobic bacteria; third, the use of natural chromophores with favorable biocompatibility profiles may reduce collateral damage compared with prolonged antiseptic use. Integration is straightforward: after mechanical biofilm disruption and irrigation, apply photosensitizer, maintain a fixed incubation and standardized rinse, then illuminate with controlled fluence using flat-top optics to cover thread geometry. Success in vivo should be judged against clinically meaningful endpoints such as bleeding on probing reduction, pocket depth change, suppuration resolution, and radiographic bone level stabilization, with CFU or quantitative PCR from explanted tips used as translational microbiologic surrogates. While blue light has shallower penetration than red or near-infrared systems and the formulation showed some dark activity, these constraints can be managed by limiting use to exposed or surgically accessed surfaces and by optimizing incubation, rinsing, and fluence within the operative field. In short, for cases where conventional antibacterial strategies underperform or where chlorhexidine tolerance is a concern, a dual-photosensitizer, blue-light aPDT step offers a plausible, implementable adjunct to improve local decontamination of implant surfaces and may help reduce recurrence risk when combined with meticulous mechanical therapy and risk factor control.

### 4.3. Limitations of This Study

This study has several important limitations. First, the model employed a single-species biofilm of *Candida albicans*, whereas clinical peri-implantitis is polymicrobial, involving anaerobic bacteria and interkingdom interactions that drive disease progression. Although a single-species system ensured standardized conditions and clear antifungal readouts, it does not reflect the complexity of clinical microbiota, and incorporating strict anaerobes in vitro remains technically challenging because of their oxygen sensitivity. All experiments were conducted in vitro, without consideration of host immunity, saliva, or peri-implant soft tissue architecture, all of which influence biofilm behavior and treatment response. These factors restrict translational inference, underscoring the need for future work using multispecies biofilms, animal models, and ultimately clinical trials. Mechanistic assessment was also limited: drug release, cellular uptake, ROS generation, and apoptosis-like responses were not directly measured, as no fluorescence microscopy, flow cytometry, ROS probes, or apoptosis assays were performed on *Candida* biofilms grown on titanium. Consequently, mechanistic interpretations remain speculative and require validation with uptake imaging, ROS quantification, and apoptosis detection. Our use of a 450 nm diode laser was guided by the absorption maxima of curcumin (~420–430 nm) and riboflavin (~445 nm), yet blue light scatters more readily in tissues than longer wavelengths (>600 nm), which are clinically preferable for deeper penetration. Moreover, subsequent studies should compare this formulation with established, longer-wavelength photosensitizers that already have regulatory approval. Light-only (L+P−) and photosensitizer-only (L−P+) groups showed limited antifungal activity, with the modest effect of light alone consistent with ROS generation from endogenous porphyrins and flavins under blue illumination [46,47,48], and the moderate effect of photosensitizer alone likely reflecting the known dark bioactivity of curcumin and riboflavin, which can disrupt ergosterol synthesis, ATPase activity, and membrane integrity even without irradiation [40,41,42,43,44,45]. Regarding outcome measures, colony-forming unit counts were employed as the primary and most reliable indicator of fungal survival and antifungal efficacy. This approach provides a direct measure of viable microorganisms and is widely regarded as the gold-standard endpoint in microbiological studies. Finally, reproducibility is constrained by the absence of full compositional details for QroxB2, as the manufacturer does not disclose the relative proportions of curcumin, riboflavin, or other formulation parameters, complicating comparisons with other photosensitizers. CFU enumeration was employed as the sole endpoint for assessing fungal viability, providing direct but limited insight into long-term survival mechanisms. The lack of complementary assays, such as clonogenic survival, ROS quantification, or uptake studies, restricts mechanistic resolution and should be addressed in future work. Future research should therefore focus on fully characterized formulations, mechanistic validation, and clinically optimized light parameters to better define the translational potential of this dual-photosensitizer strategy. Curcumin, a key component of QroxB2, is known to be unstable in aqueous solution due to photodegradation and pH sensitivity. This instability could affect therapeutic consistency and limit clinical reliability. In the present study, this risk was mitigated by reconstituting the lyophilized powder immediately before each application and maintaining dark incubation until irradiation. Nevertheless, future work should include systematic evaluation of QroxB2 stability under different storage and application conditions, providing quantitative data to confirm its suitability for clinical use. Although chlorhexidine eradicated all viable cells in this study, its cytotoxicity, interference with wound healing, and potential incompatibility with peri-implant tissues limit its clinical applicability. In contrast, the blue-light–activated natural chromophores used here, curcumin, riboflavin, and especially their combination in QroxB2, demonstrated biofilm reduction while offering a potentially greater biocompatibility profile and can be activated with spatial precision, reducing off-target effects. These features represent potential advantages for QroxB2 as an adjunctive therapy, even if absolute antifungal efficacy remains lower than that of chlorhexidine. We could not report the exact mass concentration of QroxB2 because the manufacturer does not disclose the fill mass or component ratios; we therefore standardized by optical density and dose per surface area, which we acknowledge as a limitation for absolute dose replication.

### 4.4. Implications and Translational Potential

This study demonstrates that photodynamic therapy with the curcumin–riboflavin mixture achieved a marked reduction in *Candida albicans* biofilms on titanium implant surfaces, providing foundational evidence for light-activated antifungal strategies in peri-implantitis. The significant suppression of fungal viability observed here suggests potential clinical value as an adjunct where standard mechanical or chemical decontamination is insufficient. However, translation requires further validation in multispecies biofilm models and in vivo systems that incorporate host immune responses and peri-implant tissue interactions. Several study-specific limitations should be noted. The photosensitizer mixture absorbs only in the blue region (<500 nm), necessitating relatively high fluence and limiting tissue penetration compared with red or near-infrared photosensitizers. Moreover, moderate dark toxicity was observed with the photosensitizers alone, consistent with the known bioactivity of curcumin and riboflavin, but highlighting the lack of complete selectivity; an ideal photosensitizer would be inert without light. The exact composition of the formulation remains proprietary, which limits understanding and reproducibility. In addition, only one laser setting (450 nm, 400 mW, 120 s) was tested; although these parameters were based on prior optimization [37], broader evaluation of fluence and exposure times is warranted. Outcome measures were also limited: CFU enumeration was employed as the primary endpoint for antifungal efficacy, but no complementary survival or viability assays were included. Future studies should incorporate additional methods, such as clonogenic survival or direct viability staining, to provide deeper mechanistic insight and more robust validation of treatment efficacy. Finally, photodynamic therapy as a modality carries general challenges, including limited light penetration, reduced efficacy against mature biofilms compared with planktonic cells, and the requirement for specialized light-delivery devices in clinical settings. These constraints underscore the need for continued research, refinement of photosensitizer systems, and rigorous clinical testing before photodynamic therapy can be integrated into standard peri-implantitis management.

### 4.5. Future Directions

Future work should benchmark the curcumin–riboflavin system against photosensitizers that absorb above 600 nm under matched fluence, illuminate titanium-relevant geometries with optimized light delivery, and expand to multispecies biofilms that include strict anaerobes, followed by in vivo peri-implant models. Mechanistic studies should quantify uptake, reactive oxygen species, and cell death pathways using labeled photosensitizers, dedicated ROS probes, and apoptosis assays, and relate these readouts to clonogenic survival, which should remain the primary endpoint. Formulation studies should fully characterize particle size distribution, polydispersity, ζ-potential stability, and release kinetics in saliva-mimicking media, minimize dark toxicity, and assess batch-to-batch variability. Parameter optimization should systematically vary wavelength, fluence, irradiance, exposure time, and pre-irradiation incubation, including rinsing protocols, to define therapeutic windows. Translation should address safety and regulatory requirements for dental use, including cytotoxicity to peri-implant tissues, thermal rise, device usability in the sulcus, and sterilizable applicators, and progress to well-powered randomized clinical trials that test aPDT as an adjunct to mechanical debridement with predefined clinical outcomes.

## 5. Conclusions

In this in vitro model, a dual curcumin riboflavin photosensitizer activated with 450 nm light substantially reduced *Candida albicans* biofilm on implant-grade titanium. aPDT lowered viable counts by a median of 94 percent relative to untreated controls. Chlorhexidine eliminated all recoverable cells but remained limited by known biocompatibility concerns. Light alone showed no antifungal effect and the photosensitizers alone produced a moderate decrease, which supports a light-dependent mechanism as the main driver of efficacy. These findings position blue light aPDT with natural chromophores as a practical adjunct to mechanical debridement rather than a replacement for established disinfection. The protocol used a chairside-compatible wavelength and standardized incubation and rinse steps, which supports near-term translational work. Interpretation should be tempered by study constraints. This study was limited by its single-species biofilm model, the use of in vitro conditions that exclude host factors, and the proprietary composition of QroxB2, which constrains dose comparability and mechanistic interpretation. Host cytotoxicity and tissue effects were not assessed, and blue light has limited penetration compared with longer wavelengths. Future research should test this approach in multispecies and in vivo models, quantify uptake and ROS generation, optimize fluence and exposure, and evaluate safety on peri implant tissues. Well-designed clinical trials that add aPDT to standard peri-implant therapy and use predefined clinical endpoints will be required to determine whether the observed antifungal effect translates into better healing and lower recurrence in patients.

## Figures and Tables

**Table 1:** Total yeast load in Colony Forming Units per milliliter (CFU/mL) in the different study groups. GC—Growth Control; CHX—Chlorhexidine; RIB—Xlinker Gel (riboflavin 0.1%); CUR—Curcumin-Gel 95+; QBX—QroxB2; L—Laser irradiation only; L + RIB—Xlinker Gel + Laser (riboflavin-mediated antimicrobial photodynamic therapy); L + CUR—Curcumin-Gel 95+ + Laser (curcumin-mediated antimicrobial photodynamic therapy); L + QBX—QroxB2 + Laser (QroxB2-mediated antimicrobial photodynamic therapy).

Group	n	Mean	Standard Deviation	Median	Minimum	Maximum	Interquartile Range
GC	7	44,085.7	20,062.9	49,000	23,600	71,000	38,100
CHX	7	0.0	0.0	0	0	0	0
RIB	7	44,814.3	20,505.2	45,100	21,700	75,200	37,300
CUR	7	34,957.1	21,466.2	32,700	10,300	60,700	48,500
QBX	7	26,814.3	26,088.8	21,800	8300	84,000	15,500
L	7	42,257.1	21,465.0	49,000	13,300	69,000	38,000
L + RIB	7	32,935.7	11,377.1	33,000	10,850	45,500	14,000
L + CUR	7	8807.1	2687.7	9850	3100	11,500	1300
L + QBX	7	2828.6	1398.5	2800	1500	5000	2800

## Data Availability

The raw data supporting the conclusions of this article will be made available by the authors on request.
